# Validation of the Updated (March 2025) Modified Checklist for Autism in Toddlers, Revised, with Follow-Up (M-CHAT-R/F) in Greek

**DOI:** 10.3390/children13050606

**Published:** 2026-04-27

**Authors:** Flora Bacopoulou, Dimitrios Dimitriou, Diana L. Robins, Antonios I. Christou, Theodora Xenopoulou, Christos Prapas, Vasiliki Efthymiou

**Affiliations:** 1 Clinic for Assessment of Learning Difficulties, Center for Adolescent Medicine and UNESCO Chair in Adolescent Health Care, First Department of Pediatrics, Medical School, National and Kapodistrian University of Athens, Aghia Sophia Children’s Hospital, 1 Thivon Street, 11527 Athens, Greece; 2 Cyprus Developmental and Behavioral Pediatrics Society, 1083 Nicosia, Cyprus; drdimi@yahoo.com; 3 AJ Drexel Autism Institute, Drexel University, Philadelphia, PA 19104-3734, USA; drobins@drexel.edu; 4 Department of Special Education, University of Thessaly, 38221 Volos, Greece; antchristou@uth.gr; 5 Developmental and Behavioral Pediatric Clinic, General Hospital of Nikaia—Piraeus “St. Panteleimon”, 18454 Athens, Greece; doxe.gr@gmail.com; 6 Department of Public and Community Health, School of Public Health, University of West Attica, 11521 Athens, Greece

**Keywords:** autism, M-CHAT-R/F, autism spectrum disorder, Greek, M-CHAT, screening, validation, developmental disabilities, developmental disorders

## Abstract

**Highlights:**

**What are the main findings?**

- The Greek version of the M-CHAT-R/F (terminology update March 2025) demonstrated good reliability and acceptable scalability.
- The M-CHAT-R/F had strong diagnostic accuracy in a Greek sample of children aged 16–48 months.

**What are the implications of the main findings?**

- The Greek M-CHAT-R/F is a culturally appropriate screening tool for the autism spectrum disorder (ASD) risk in routine pediatric practice in Greece.
- Its strong diagnostic performance supports its integration into nationwide early childhood health programs, facilitating timely referral and early intervention for children with autism in Greece.

**Abstract:**

**Background/Objectives**: The aim of this study was to translate, culturally adapt, and validate the updated (March 2025) version of the Modified Checklist for Autism in Toddlers, Revised, with Follow-Up (M-CHAT-R/F) in Greek by examining its reliability, validity, and screening efficacy in a Greek sample. **Methods**: The M-CHAT-R/F was administered to Greek parents/primary caregivers of children aged 16–48 months, with no prior diagnosis of autism spectrum disorder (ASD), severe sensory impairments, or other chronic conditions causing neurodevelopmental delay. Parents/primary caregivers were invited to participate in the nationwide Program “Child and Family Health Promotion through the Paediatric Framework” of the Greek Ministry of Health in collaboration with UNICEF, implemented by the National and Kapodistrian University of Athens. **Results**: The final sample comprised 280 parents of children 16 to 48 months old (mean age ± SD 24.64 ± 7.75 months) with an almost equal sex distribution. Mean (±SD) parental age was 35.31 (±3.14) years, ranging from 30 to 48 years, and most respondents were mothers (94.3%). The Greek M-CHAT-R/F demonstrated acceptable reliability (KR-20 = 0.789) and acceptable scalability (monotonicity). **Conclusions**: The present study provides compelling evidence that the Greek M-CHAT-R/F is a reliable, valid, culturally appropriate instrument for screening children for the presence of ASD. The psychometric properties of the Greek version are consistent with previous international reports and warrant its use in routine pediatric practice in Greece.

## 1. Introduction

Autism Spectrum Disorder (ASD) is an intricate neurodevelopmental disorder characterized by difficulties in social communication and interaction, as well as repetitive behavior(s), interests, or activities [1]. The early indications of ASD frequently occur during the early childhood years (infant and toddler) usually before the age of 3; however, due to the subtlety of the initial behaviors or lack of available resources to perform developmental screening, diagnosis may occur many months or even years later [2]. Timely identification of ASD is essential because there has been a consistent connection between early intervention and improved developmental outcomes, social skills, and overall long-term positive outcomes for children with ASD and their families [3].

The global incidence of ASD has steadily increased in recent years; estimates of the number of children with ASD have recently ranged between 1 in 31 to 1 in 100 children in different countries due to differences in diagnostic criteria, methodologies, and reporting practices for healthcare systems [4,5,6]. These increases demonstrate a need for dependable and validated screening tools for the successful identification of early signs of ASD across numerous cultures and languages. The implementation of screening instruments allows for referral and diagnosis to occur in a timely manner. In addition, these early tools may assist healthcare providers in determining how to best allocate resources to meet identified needs or develop evidence-based intervention services. By accessing diagnosis at an earlier age than has been feasible in the past, children will receive optimal developmental support.

A widely used early screening tool developed for detection of ASD is the Modified Checklist for Autism in Toddlers, Revised, with Follow-Up (M-CHAT-R/F) [7]. The M-CHAT-R/F, which is designed for children aged 16–30 months, is an updated version of the original Modified Checklist for Autism in Toddlers (M-CHAT). The M-CHAT-R/F includes a questionnaire for parents and an additional structured interview that a trained interviewer administers to parents of children who score in the elevated likelihood of autism range, ensuring a low level of false positive results and an increased level of specificity for detection of ASD [7]. Multiple studies have examined the tool’s strong psychometric properties, clinical utility, and efficiency for use in both primary and community care settings [8] to screen children up to the age of 48 months. Consequently, the M-CHAT-R/F has been adopted as a standard first-line screening tool for children 16 to 48 months old throughout a variety of healthcare systems.

Although the M-CHAT-R/F is widely available throughout the world, it is essential that the reliability and validity of the M-CHAT-R/F is established individually for every cultural/linguistic setting, as cultural differences, different views of how parents see growth and development, and how parents educate their children all influence how ASD behaviors are perceived and reported [9]. When adapting M-CHAT-R/F for cross-cultural use, the focus is not simply on translating M-CHAT-R/F from its original language to another language; it also ensures that M-CHAT-R/F has the same conceptual framework, respect for the culture, and psychometrically sound evaluation that support the correctness of diagnosing non-English-speaking children as if they were diagnosed in English-speaking populations [10]. Without providing evidence that this validation process has been completed prior to administering the M-CHAT-R/F, individuals may misinterpret screen results for children, which may delay the diagnosis of ASD. Furthermore, inappropriately administered or scored screening may prevent children from receiving timely access to early intervention services because of a lack of accurate screening results.

Although public knowledge of ASD and access to diagnostic services are improving in Greece, many professionals are not yet using standardized screening measures consistently to diagnose children under two years of age. In Greece, the diagnosis of ASD depends on the expertise of health professionals in clinical evaluation, namely, developmental pediatricians, child psychiatrists, or child neurologists. The ICD-10 clinical criteria, as formulated in the International Classification of Diseases of the World Health Organization [11], are used for coding the cases in the Greek social insurance system [12,13]. At present, there are not any recommended universal autism screening tools in Greece. The gold standard tools Autism Diagnostic Observation Schedule (ADOS-2) and the Autism Diagnostic Interview-Revised (ADI-R) are known to be employed by some professionals; however, they are not essential for ASD diagnosis, they are neither officially translated nor validated in the Greek setting, and the extent to which these tools are applied is unknown [13].

Therefore, there has been limited investigation into the use of ASD screening measures for toddlers within the Greek context. To this date, there has been no validation of the M-CHAT-R/F in Greek; a recent study [14] reported failing monotonicity of a previous M-CHAT-R/F Greek translation and suggested that further validation is essential to confirm its psychometric properties in the Greek context.

Consequently, there is a noticeable gap between the clinical recommendations from the World Health Organization (WHO) and how professionals in Greece use M-CHAT-R/F. To provide pediatricians, child psychologists, speech–language pathologists, and professionals who provide early intervention services with a psychometrically sound tool for identifying which toddlers might need a more comprehensive assessment of their development, it is critical to produce robust psychometric data regarding the M-CHAT-R/F in Greece.

Validation in Greek is necessary, as M-CHAT-R/F items are behaviorally subtle, and scores may be systematically biased. Furthermore, some M-CHAT-R/F items are culture sensitive, and item monotonicity and predictive value can change, and therefore psychometric values must be re-established. Especially the Follow-Up interview is language dependent, and using a non-validated version bears risks, whereas validation ensures equal screening quality.

Thus, the primary aim of this study was to translate, culturally adapt, and validate the updated (March 2025) version of M-CHAT-R/F in Greek by examining the tool’s reliability, preliminary validity, and screening efficacy in a Greek sample. A secondary aim was to compare the psychometric properties of the M-CHAT-R/F to those already reported in other countries and investigate the feasibility of implementing M-CHAT-R/F into routine primary screening programs. The establishment of normative data and diagnostic performance characteristics in the Greek context is anticipated to greatly enhance early ASD detection systems in Greece and improve the ability of healthcare providers and clinicians to make informed clinical decisions, which should ultimately improve the developmental outcomes for children and families in Greece.

## 2. Materials and Methods

### 2.1. Study Design

This cross-sectional validation study evaluated the psychometric properties of the M-CHAT-R/F in a Greek sample. This study comprised three phases (i) linguistic translation and cultural adaptation, (ii) pilot testing for clarity and comprehension, and (iii) large-scale administration to assess reliability, validity, and screening accuracy.

### 2.2. Participants

Participants were Greek parents/primary caregivers of children aged 16–48 months, who had no prior diagnosis of ASD, severe sensory impairments (i.e., profound hearing or vision loss), or other known neurological, metabolic, or other chronic conditions causing neurodevelopmental delay, unrelated to ASD.

Parents/primary caregivers were invited, while attending health checks, from October 2025 to January 2026, to participate in the nationwide Program “Child and Family Health Promotion through the Paediatric Framework” of the Greek Ministry of Health in collaboration with UNICEF, implemented by the National and Kapodistrian University of Athens. It is a program of the 1st Pillar of the National Action “Child and Family Health Promotion” within the framework of the National Recovery and Resilience Plan Greece 2.0, funded by the European Union—NextGeneration EU. The research has been approved by the research and ethics committee of the Aghia Sophia Children’s Hospital in Athens, Greece (protocol number 20798/10.9.2025) and complied with the ethical standards of the Declaration of Helsinki.

Participation was voluntary, and families could withdraw at any point without consequences. Confidentiality of all data was ensured.

### 2.3. Procedures

Parents/primary caregivers who provided informed consent completed the updated (terminology update March 2025) Greek version of the M-CHAT-R/F. Responses were scored according to the official scoring guidelines. Children screened as moderate likelihood for ASD were contacted for the structured Follow-Up interview, administered by trained pediatricians by telephone. The Follow-Up aimed to clarify item responses and reduce false-positive outcomes. Children with persistent positive screening results after the Follow-Up were referred for comprehensive clinical evaluation to developmental or mental health services.

#### 2.3.1. M-CHAT-R/F

The M-CHAT-R/F identifies the earliest signs of ASD in children between 16 and 30 months old [7]; several studies have demonstrated utility up to 48 months [15,16,17,18,19]. The tool comprises two sequential stages; the M-CHAT-R (first stage) completed by parents and a Follow-Up (second stage) interview administered by professionals exclusively to parents of children who scored in the “moderate-likelihood” range on the M-CHAT-R. The primary goal of the M-CHAT-R is to maximize sensitivity, i.e., to detect as many cases of ASD as possible, whereas the purpose of the Follow-Up is to reduce false positives among children in this moderate-likelihood range—the “grey zone”—by clarifying ambiguous responses through a structured parental interview. The M-CHAT-R consists of 20 questions that can be answered with a Yes/No from parents/primary caregivers, and the questions address social communication, joint attention, imitation skills, play behaviors, and how children respond to social stimuli. Each question assesses a behavior that might indicate ASD or other developmental delay. Yes/No responses to each item of the M-CHAT-R are replaced by 0/1 on the M-CHAT-R/F Scoring Sheet, and the overall score categorizes children into low, moderate, or high likelihood of autism. Children with a high likelihood of ASD (score 8–20) are referred immediately for clinical evaluation. For children with a moderate likelihood of ASD (score 3–7), a structured Follow-Up (F) is conducted to help clarify their responses, decrease the rate of false positives, and increase the specificity of the tool, thereby improving its diagnostic performance [7]. If the score on Follow-Up is 0–1, children have screened negative and no further action is required unless clinical surveillance indicates elevated likelihood for ASD. If children screen positive for ASD on Follow-Up (high-likelihood score ≥2), they are referred for clinical evaluation and early intervention as soon as possible. The M-CHAT-R/F has demonstrated strong psychometric properties in various studies, including high sensitivity to early social-communication deficits, strong screening utility in primary care settings, and a reduction in age of referral for diagnostic evaluation [20,21]. In March 2025, the M-CHAT-R/F creators released an update that adjusts terminology with no changes in instructions, items, or scoring.

#### 2.3.2. Translation and Cultural Adaptation

The M-CHAT-R/F was translated into Greek following permission of one of its creators, Professor Diana L. Robins. The procedure included forward and back-translation according to internationally recognized guidelines for cross-cultural adaptation of psychometric instruments and comprised four stages. In forward translation, two independent bilingual experts translated the original English version into Greek. Then, the supervisor of the study synthesized the two versions and merged them into a single preliminary version. Back-translation included a separate pair of bilingual translators, blinded to the original instrument, who translated the Greek version back into English to verify conceptual equivalence. An expert panel consisting of psychologists, child and adolescent psychiatrists, pediatricians, and developmental pediatricians reviewed all versions to ensure cultural relevance, semantic accuracy, and clarity. Lastly, the pre-final version was administered to a pilot group of parents (*n* = 20) to assess readability and comprehension. Following this process, minor linguistic modifications were applied to the 9th and 19th items to improve clarity and cultural appropriateness while preserving the conceptual content of the original items (linguistic transformations). Specifically, item 9 was linguistically rephrased to better reflect common Greek caregiving terminology (as there is no infinitive in Greek language), whereas in item 19, an explanatory term was added to better reflect the original meaning. No items were omitted or substantively altered in meaning.

### 2.4. Statistical Analysis

All statistical analyses were performed using the IBM SPSS Statistics version 29 (IBM Corp. Released 2023. IBM SPSS Statistics for Windows, Version 29.0.2.0; Armonk, NY, USA: IBM Corp) and R Statistics software version 4.5.2 [22] using the Mokken package [23]. No item-level missing data were observed, as full completion of the M-CHAT-R/F was required for scoring. Continuous variables are expressed as mean ± standard deviation (SD). Internal consistency was assessed using Kuder–Richardson Formula 20 (KR-20). ASD was diagnosed by developmental pediatricians and/or child psychiatrists. Item scalability and monotonicity were evaluated using non-parametric Mokken scaling, which is appropriate for heterogeneous screening instruments. Non-parametric Mokken scaling was conducted in preference to parametric IRT models, given the dichotomous response format of the items and the small number of confirmed ASD cases in the study sample. Significance level was set at *p* < 0.05.

## 3. Results

### 3.1. Study Sample Characteristics

Parents of 280 children were initially enrolled in the study. Of these, 73 (26%) completed both the initial questionnaire and the Follow-Up as indicated (children whose initial M-CHAT-R score was 3–7). The demographic characteristics of the final study sample (*N* = 280) are presented in Table 1. Most respondents were mothers (94.3%), while fathers accounted for 5.7% of the parents. Regarding children’s sex, 53.2% were male and 46.8% were female, indicating an almost equal gender distribution. Mean parental age was 35.31 years (SD = 3.14), ranging from 30 to 48 years, whereas the mean age of the children was 24.64 months (SD = 7.75), ranging from 16 to 48 months.

As shown in Table 2, when comparing the results obtained from both parts of the tool, the Greek M-CHAT-R and the Greek M-CHAT-R/F, it is evident that the distributions of ASD likelihood categories differ slightly. Following assessment with the Greek M-CHAT-R, 72.8% of children were classified as “low likelihood”, 26.1% were classified as “moderate likelihood”, and 1.1% were classified as “high likelihood” of ASD, based on the 0–2, 3–7, 8–20 total score responses, respectively. After conducting structured Follow-Up of the 73 children in the “moderate likelihood” range, 58 (79.5%) were finally classified in the “low likelihood” category, whereas 15 (20.5%) were classified in the “high likelihood” range. Among the 18 cases of high likelihood (3 + 15), referred for clinical evaluation to developmental pediatricians and/or child psychiatrists, 4 children were diagnosed with ASD (1.4%), yielding a positive predictive value (PPV) of 22.2% (95% CI: [6.4%, 47.6%]).

### 3.2. Reliability

The Greek version of the M-CHAT-R/F has high reliability, with a KR-20 value of 0.789 for the internal consistency of the 20 items of the questionnaire. Also, the Greek version of the M-CHAT-R had a reliability score of 0.812 (95% CI: 0.761–0.853).

Non-parametric Mokken scaling was conducted to examine item scalability and monotonicity. Items with zero response variance were excluded from these analyses. The remaining items formed a strong Mokken scale, with an overall scalability coefficient of H = 0.76 (standard error; SE = 0.05), indicating high hierarchical consistency. Individual item scalability coefficients were generally above the recommended threshold for screening instruments. Monotonicity assumptions were met for all items, with no statistically significant violations observed, indicating that the probability of item failure increased consistently with increasing total risk score.

## 4. Discussion

The goal of this study was to translate, culturally adapt, and validate the Greek version of the M-CHAT-R/F by examining its psychometric properties in a Greek sample of parents of children aged between 16 and 48 months. The findings of this study support the reliability and validity of the Greek M-CHAT-R/F as an effective screening tool for early identification of ASD and are consistent with the results of international validation studies.

A direct comparison between the updated Greek version of the initial M-CHAT-R and the two-stage M-CHAT-R/F indicated that the structured Follow-Up reduced the screen positive rate by reverting some children who were originally above the threshold of 3+ items to the low-likelihood group. This pattern is consistent with previous validation where there was a decrease in the false-positive rate as a result of clarifying parent responses and providing context for the behaviors of children who may be misinterpreted as demonstrating elevated likelihood of ASD [7].

The prevalence of ASD detected in the present study sample was 1.43% (4 out of 280 children), a finding consistent with current global epidemiological estimates. As noted earlier, reported prevalence rates range from 1 in 31 to 1 in 100 children across different countries, reflecting variability in diagnostic criteria, methodologies, and healthcare reporting practices [4,5,6]. Given this range, the rate identified in the present sample falls within expected epidemiological bounds. Previous epidemiological data from Greece, derived from only one nationwide study in 2019, estimated ASD prevalence in a large population of 182,879 schoolchildren aged 10 and 11 years, using administrative data from the public Centers for Multidisciplinary Assessment, Counseling and Support (KEDASY). The overall prevalence of ASD over 13 regions across the country was 1.15%, ranging from 0.59% to 1.50% (1.83% in males; 0.44% in females; male-to-female ratio 4.14:1) [13]. Furthermore, as the M-CHAT-R/F is a level 1 screening tool designed for use in community settings (where the base rate of ASD is inherently low) the number of positive cases identified is methodologically expected and does not compromise the validity of the screening procedure. These findings support the appropriateness of adaptation of the M-CHAT-R/F in the Greek setting.

The decreased number of children who screened positive after the Follow-Up illustrates the role of the two-stage screening method, specifically in those cultures where parents have differing interpretations of child development. The findings suggest that the M-CHAT-R/F has clinical utility in promoting efficient and accurate screening in routine pediatric environments, allowing healthcare providers to make timely referrals for diagnosis.

The internal consistency of the Greek version of M-CHAT-R/F appears to be adequate (KR-20 = 0.789), reflecting its acceptable reliability as a screening tool for early detection of ASD. The reported internal consistency for the Greek updated version appears to be consistent with the reported internal consistency for other language versions, including the English version and other international validity studies at a reliability score range of 0.70 to 0.80 [5,6,7,20]. Mokken scaling results demonstrated acceptable monotonicity and scalability, supporting the construct validity of the measure despite moderate KR-20.

The successful process of translating and adapting the M-CHAT-R/F in Greek indicates that it is likely to be applicable in a wide variety of cultures if the process is followed with rigorous methodology. Minor linguistic changes applied to adapt the items were deemed indispensable to maintain clarity of meaning and cultural validity, consistent with previous findings on the importance of considering culture when interpreting parental reports of their children’s early developmental behavior [9].

These findings highlight the fact that effective adaptation requires an understanding of how cultures recognize aspects of development as well as how parents (and caregivers) understand and report about child development. The psychometric qualities of the Greek M-CHAT-R/F demonstrate that cultural differences and norms surrounding child development, communication, and social behavior have all been taken into account when adopting the instrument in the Greek context.

In addition to creating a new screening instrument in Greek, the validation of the updated M-CHAT-R/F fills an important need for a psychometrically sound screening tool that is developed according to international clinical practice guidelines. The availability of such a tool has significant implications for pediatricians, child psychologists, speech–language pathologists, early intervention providers, etc. Routine use of the M-CHAT-R/F in Greece could help identify children with ASD sooner and lead to quicker referral for diagnosis and intervention, which has consistently been found to result in better developmental outcomes [2,3]. Implementing a standardized screening tool could also result in equitable access to early identification services and a more efficient use of available healthcare resources at the public health level.

There are some limitations to be considered in this study. The study sample was relatively small and predominantly drawn from the Athens metropolitan area; however, participants included Greek-speaking inhabitants of several rural (approximately 23%) or urban (approximately 77%) areas around Greece. Cross-cultural adaptation of tools for autism screening and diagnosis requires representation from the target culture to validate an instrument in the “target language” that is conceptually equivalent to the original, rather than strict demographic representativeness in a nationally representative sample [24].

Additionally, most respondents were mothers (94.3%), which may introduce a reporter bias, as paternal perspectives and observations were largely absent. However, in Greece, mothers are usually the primary caregivers for children of such small ages. Another limitation pertains to the absence of standardized instruments such as the ADOS-2 or the ADI-R for diagnostic confirmation of identified cases, which have not been validated in Greek. Moreover, due to the absence of diagnostic follow-up for screen-negative children, sensitivity, specificity, and negative predictive value could not be estimated. Consequently, ROC/AUC analysis was not performed, as it requires a fully verified sample with known diagnostic status across all screening outcomes as well as larger number of true positives. These constraints are inherent to level 1 community screening designs and are consistent with limitations reported in other community-based M-CHAT-R/F validation studies [4,6,8]. Furthermore, as study participants were recruited through a national child health program, refusal rates were not formally tracked, preventing comparison of demographics between participants and non-participants. This study utilized a cross-sectional design, whereas a longitudinal follow-up would allow for determining the predictive validity of developmental outcomes. Future studies should utilize larger, more heterogeneous samples from geographically diverse regions of Greece with more balanced parental representation and incorporate gold-standard diagnostic tools to strengthen the validity of the Greek M-CHAT-R/F adaptation. Also, future research should ideally incorporate a randomly selected subsample of screen-negative children for clinical evaluation in order to provide unbiased estimates of sensitivity and NPV and to enable robust ROC analysis.

## 5. Conclusions

The present study provides compelling evidence that the Greek M-CHAT-R/F is a reliable, valid, culturally appropriate instrument for screening children for the presence of ASD. The psychometric properties of the Greek version are consistent with previous international reports and warrant its use in routine pediatric practice in Greece. The Greek M-CHAT-R/F has the potential to greatly improve the rate of early detection of autism and the developmental outcomes of affected children and their families. Further research should assess the feasibility of incorporating the Greek M-CHAT-R/F into universal primary care screening in Greece and identify the obstacles faced by healthcare professionals in delivering effective screening.

## Figures and Tables

**Table 1:** Demographic characteristics of the study sample (*N* = 280).

	n	%
Parent		
Father	16	5.7
Mother	264	94.3
Child’s sex		
Male	149	53.2
Female	131	46.8
Parental age (years) *	35.31 ± 3.14 (30–48)	
Child’s age (months) *	24.64 ± 7.75 (16–48)	

Notes. Values refer to absolute (n) and relative frequency (%) or * mean ± standard deviation.

**Table 2:** Comparison of the screening procedures using the Greek M-CHAT-R vs. M-CHAT-R/F.

	n	%
Greek M-CHAT-R	280	
Low likelihood: 0–2	204	72.8
Moderate likelihood: 3–7	73	26.1
High likelihood: 8–20	3	1.1
Greek M-CHAT-R/F	73	
Low likelihood: 0–1	58	79.5
High likelihood: ≥2	15	20.5

Notes. Values refer to absolute (n) and relative frequency (%).

## Data Availability

The data presented in this study are available upon request from the corresponding author due to privacy restrictions.

## Abbreviations

The following abbreviations are used in this manuscript:

ASD	Autism Spectrum Disorder
ADI-R	Autism Diagnostic Interview-Revised
ADOS-2	Autism Diagnostic Observation Schedule
ICD-10	International Classification of Diseases, 10th Revision
M-CHAT	Modified Checklist for Autism in Toddlers
M-CHAT-R	Modified Checklist for Autism in Toddlers, Revised
M-CHAT-R/F	Modified Checklist for Autism in Toddlers, Revised, with Follow-Up
WHO	World Health Organization
SD	Standard Deviation
KR-20	Kuder–Richardson Formula 20
CI	Confidence Intervals
